# Dataset on tip vortex formation noise produced by wall-mounted finite airfoils with flat and rounded tip geometries

**DOI:** 10.1016/j.dib.2019.105058

**Published:** 2019-12-31

**Authors:** Tingyi Zhang, Danielle Moreau, Thomas Geyer, Jeoffrey Fischer, Con Doolan

**Affiliations:** a The University of New South Wales, Sydney, NSW, 2052, Australia; b Brandenburg University of Technology Cottbus—Senftenberg, 03046, Cottbus, Germany

**Keywords:** Aeroacoustics, Airfoil noise, Beamforming

## Abstract

The vortex generated at the tip of an airfoil such as an aircraft wing, wind turbine blade, submarine fin or propeller blade can dominate its wake and be a significant source of unwanted noise. The data collection presented in this paper consists of measurements of tip vortex formation noise produced by finite length airfoils with flat and rounded tips. These data were obtained using the specialist aeroacoustic test facilities at the Brandenburg University of Technology (BTU) in Cottbus, Germany and a 47-channel planar microphone array. Over 1200 unique test cases with variations in airfoil profile shape, tip geometry, angle of attack and Reynolds number were measured during the experimental campaign. The dataset contains one-third-octave band tip noise spectra that have been processed using Acoular, a Python module for acoustic beamforming.

**Table:** Specifications Table

Subject	Aerospace Engineering
Specific subject area	Acoustics, aerodynamics and fluid mechanics
Type of data	Tables in.csv format One-third-octave band acoustic spectra in.txt and.tif format
How data were acquired	Acoustic data were acquired using a planar 47-channel microphone array in an anechoic wind tunnel at the Brandenburg University of Technology in Cottbus, Germany. A National Instruments 24-bit multichannel measurement system combined with in-house software (written using a combination of Labview and Python codes) was used to record the microphone data.
Data format	Raw and analysed
Parameters for data collection	The test models were NACA 4-digit airfoils with 0–6% camber and 12–18% thickness, flat and rounded tip geometries and an aspect ratio of 2. Measurements were taken with natural and forced airfoil boundary layer transition at a wide range of Reynolds numbers (25,000 to 225,000) and geometric angles of attack (−10 to 20°).
Description of data collection	The data collection is a benchmark set of experimental measurements on wall-mounted finite airfoil tip vortex formation noise.
Data source location	The University of New South Wales, Sydney, Australia 33° 55′ 4″ S, 151° 13′ 52″ E
Data accessibility	Repository name: Mendeley data Direct URL to data: https://doi.org/10.17632/6x59x7x3ny.2

**Table:** 

**Value of the Data** • The dataset provides new information on the character of airfoil tip noise. It also gives new insight into how airfoil profile shape and tip geometry affect tip noise production. • The data can be used in the future development and validation of airfoil tip noise predictions. • The data can be used to validate computational fluid dynamic and computational aeroacoustic simulations of different airfoil tip shapes. • The data can be used in facility comparison; when compared with measurements taken in different wind tunnel facilities, the data can be used to determine whether the facility has an influence on the flow and noise results. • Students, researchers and those working in industry, who are interested in the acoustic behaviour of wall-mounted finite airfoils and specifically the wingtip, will benefit from this data collection.

## 1 Data

The data presented in this article is a benchmark set of acoustic array measurements on wall-mounted finite airfoil tip vortex formation noise. The dataset contains processed one-third-octave band sound pressure level spectra (txt and.tif format). Table 1 is a test matrix of the experimental configurations. Table 2 states the positions of the microphones in the 47-channel planar microphone array. Table 3 gives the one-third-octave band tip noise spectra for a tripped NACA0012 airfoil with flat tip at geometric angles of attack of α=0 :2.5 :15° and 20°, and a Reynolds number of $Re_{C}=2.25\times 10^{5}$, based on chord. Fig. 4 shows the tip noise spectra for tripped airfoils with flat tip at geometric angles of attack of α=0° and 15° and a Reynolds number of $Re_{C}=2.25\times 10^{5}$, based on chord. Raw and processed data for each table and figure can be accessed via the direct URL to the data: https://doi.org/10.17632/6x59x7x3ny.2.

**Table 1:** Overview of experimental configurations.

Airfoil profile	Airfoil boundary layer transition type	Tip geometry	Geometric angle of attack (°)	Reynolds Number (‘000)
NACA0012	Natural	Flat	0 : 2.5 : 15, 20	25 : 25: 225
	Forced	Flat	0 : 2.5 : 15, 20	
	Natural	Rounded	0, 5, 10, 20	
	Forced	Rounded	0, 5, 10, 20	
NACA0015	Natural	Flat	0 : 2.5 : 15, 20	25 : 25: 225
	Forced	Flat	0 : 2.5 : 15, 20	
NACA0018	Natural	Flat	0 : 2.5 : 15, 20	25 : 25: 225
	Forced	Flat	0 : 2.5 : 15, 20	
NACA2412	Natural	Flat	−10, 0 : 2.5 : 15, 20	25 : 25: 225
	Forced	Flat	−10, 0 : 2.5 : 15, 20	
NACA4412	Natural	Flat	−10, 0 : 2.5 : 15, 20	25 : 25: 225
	Forced	Flat	−10, 0 : 2.5 : 15, 20	
NACA6412	Natural	Flat	−10, 0 : 2.5 : 15, 20	25 : 25: 225
	Forced	Flat	−10, 0 : 2.5 : 15, 20	
	Natural	Rounded	0, 5, 10, 15, 20	
	Forced	Rounded	0, 5, 10, 15, 20

**Table 2:** Positions of the microphones in the beamforming array.

Microphone number	X (mm)	Y (mm)	Microphone number	X (mm)	Y (mm)	Microphone number	X (mm)	Y (mm)
0	−146	634	16	−193	−152	32	83	−42
1	−67	237	17	−120	−215	33	634	146
2	−377	530	18	−29	−244	34	237	67
3	−152	193	19	−463	−30	35	530	377
4	−551	345	20	−242	−139	36	193	152
5	−215	120	21	−88	−29	37	345	551
6	−641	108	22	−73	−269	38	120	215
7	−244	29	23	−42	−83	39	108	641
8	−30	463	24	67	−237	40	29	244
9	−139	242	25	152	−193	41	463	30
10	−29	88	26	215	−120	42	242	139
11	−349	307	27	641	−108	43	88	29
12	−269	73	28	244	−29	44	307	349
13	−83	42	29	139	−242	45	73	269
14	−634	−146	30	29	−88	46	42	83
15	−237	−67	31	269	−73

**Table 3:** One-third-octave band tip noise spectra for a tripped NACA0012 airfoil with flat tip at different geometric angles of attack and a Reynolds number based on chord of $Re_{C}=2.25\times 10^{5}$.

One-third octave band centre frequency (kHz)	One-third-octave band sound pressure level, $Lp_{1/3}$ (dB re 20 μPa)							
	0°	2.5°	5°	7.5°	10°	12.5°	15°	20°
0.50	38.51	38.37	40.10	40.13	38.74	49.07	64.57	56.32
0.63	49.73	50.44	49.79	50.24	52.44	59.47	68.80	63.42
0.79	45.88	47.23	42.73	46.20	47.99	46.57	62.15	62.66
1.00	26.85	26.47	26.95	26.14	26.33	28.97	31.05	30.34
1.26	31.77	31.56	31.80	34.04	33.43	35.00	35.27	49.20
1.58	35.63	35.58	35.93	36.48	37.83	42.06	44.31	53.81
2.00	41.35	41.61	41.94	43.28	46.12	48.87	50.04	62.55
2.51	43.25	43.81	44.52	45.53	48.60	51.07	52.31	62.82
3.16	42.75	43.03	43.32	44.34	48.16	50.63	52.95	61.81
3.98	42.13	42.49	42.64	43.62	46.30	49.18	51.99	60.54
5.01	43.74	43.72	43.63	44.09	47.06	48.87	51.47	59.29
6.31	45.12	44.98	44.88	45.27	46.65	48.40	51.04	58.03
7.94	43.37	44.04	44.40	44.82	45.54	48.21	51.21	55.83
10.00	38.65	40.77	41.32	42.82	44.93	48.74	51.44	53.25
12.59	33.21	37.46	38.70	42.50	46.70	49.92	49.37	50.21
15.85	25.47	32.14	32.88	35.68	39.84	41.22	40.31	45.70
19.95	21.63	21.43	21.97	22.14	26.12	32.10	33.60	40.59

## 2 Experimental design, materials, and methods

### 2.1 Anechoic wind tunnel facility

**Fig. 1:**
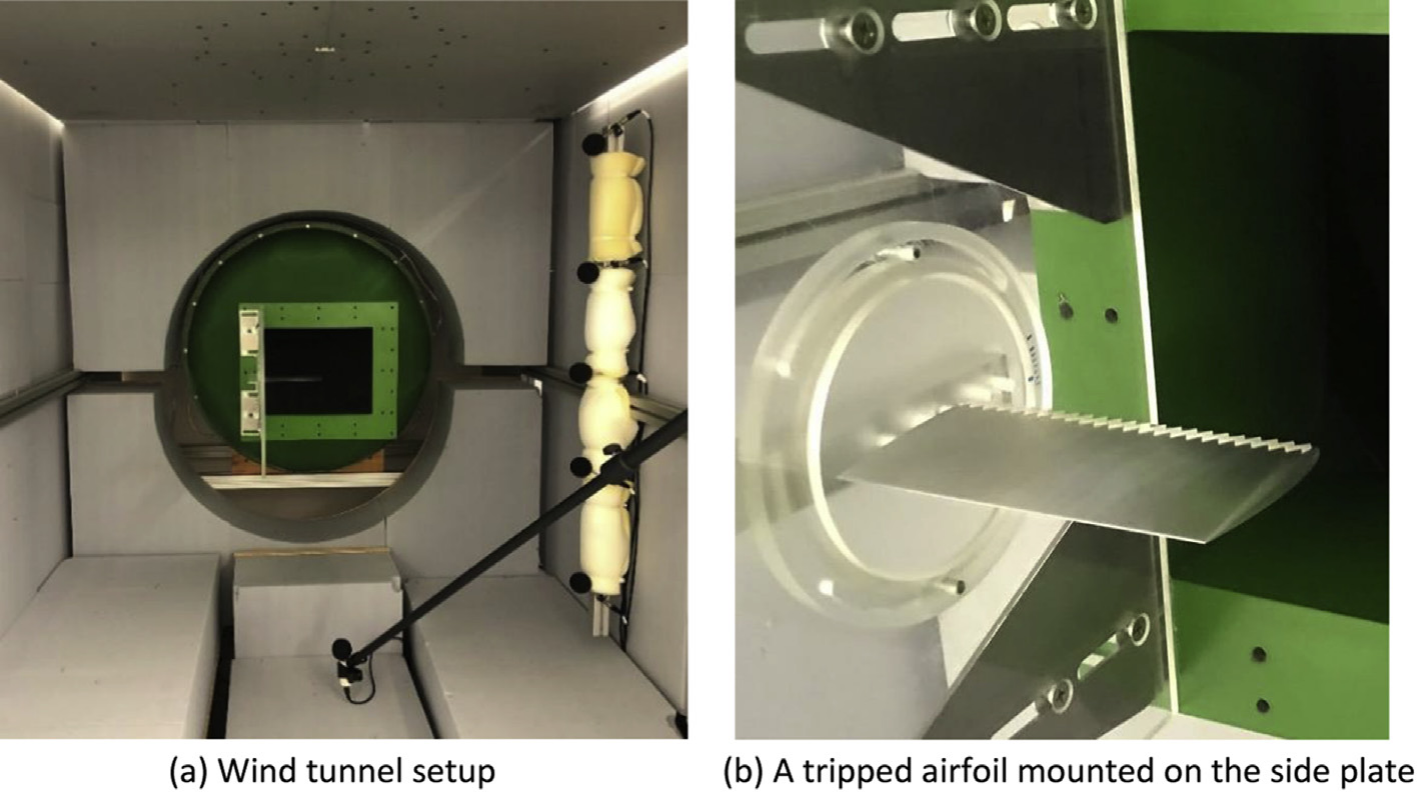
Experimental setup in the wind tunnel. The microphone array can be seen in the chamber ceiling in (a). Note that the 6 single microphones shown in (a) were not used in this experiment.

Acoustic measurements were performed in a quasi-anechoic open jet wind tunnel at the Brandenburg University of Technology in Cottbus, Germany, as shown in Fig. 1. The wind tunnel contraction outlet has a width of 280 mm and a height of 230 mm, the maximum free stream velocity of the jet is about 60 m/s and the axial turbulence intensity is less than 0.2% [1,2]. The test section is surrounded by an anechoic chamber of 1.5 m by 1.5 m in cross-section and 2.5 m in length. The chamber features absorbing walls and floor made from Basotec foam to produce a quasi-anechoic environment at frequencies over 125 Hz.

### 2.2 Test models

The test model consisted of an airfoil mounted to a side plate as shown in Fig. 1(b). The airfoils used in this dataset can be categorized into two cases: (1) NACAxx12 profile with xx = 0, 2, 4 and 6% camber at 40% chord and (2) NACA00yy profile with thickness of yy = 15 and 18% (as listed in Table 1). All airfoils have a theoretical chord length of 70 mm, an actual chord length of 67 mm due to a truncated rounded trailing edge with diameter of 1.0 mm and a span of 140 mm, corresponding to an aspect ratio of 2. One full set of airfoils was produced with a flat tip while additional airfoil models with NACA0012 and NACA6412 profile were produced with a rounded tip. The rounded tip was defined by creating semi-circles, whose diameters are equal to the local airfoil thickness, along the camber line at 135.8 mm span. All models were manufactured from aluminium using Computer Numerical Control (CNC). The airfoils were tested with both natural and forced boundary layer transition. In forced transition configuration, 60-degree zig-zag trip tape (manufactured by Glasfaser Flugzeugservice) with 0.4 mm thickness and 6 mm point-to-point distance was used on both sides of the airfoil at 10% chord.

The Perspex side plate attached to the contraction outlet (see Fig. 1(b)) is 400 mm in the streamwise direction and 360 mm in height. The airfoil is mounted to a central disc that allows rotation to adjust the airfoil angle of attack. The airfoils were mounted to the side plate one at a time and the distance between the airfoil leading edge and the contraction outlet is 104 mm at zero angle of attack.

### 2.3 Microphone array

**Fig. 2:**
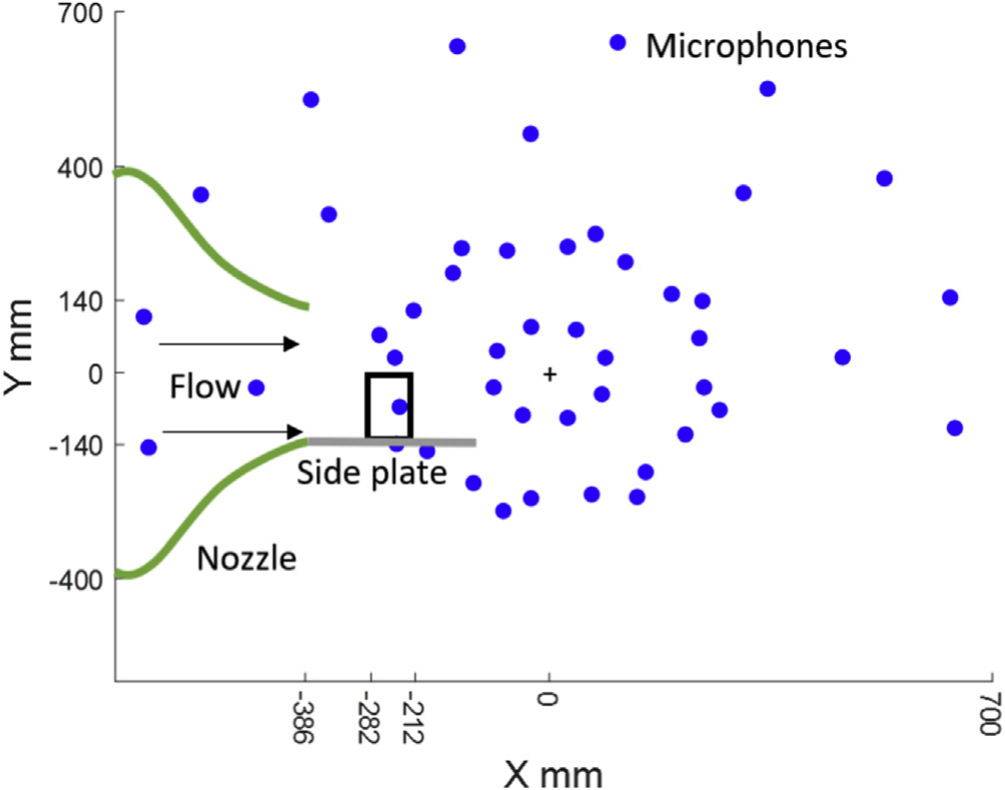
Schematic diagram of the nozzle, airfoil model (shown as a black rectangle) and planar microphone array [3].

Acoustic data were obtained using a planar microphone array containing 47 1/4-inch Panasonic microphone capsules (WM-61A), with a frequency range of 20 to 16,000 Hz, flush-mounted in the chamber ceiling 710 mm above the airfoil trailing edge at zero angle of attack, as shown in Fig. 2. The origin of the coordinate system is located at the centre of the array with X and Y being the streamwise and spanwise directions, respectively. The array focusing distance in the Z direction (normal to the plane shown in Fig. 2) was fixed to be 710 mm for all tests. The positions of the 47 microphones are given in Table 2. The microphones are numbered from 0 to 46 in order to maintain consistency with the Python syntax.

Acoustic array measurements were recorded for 40 seconds with a National Instruments 24-bit multichannel measurement system, including PXI-4472 cards in a NI PXI-1044 chassis, at a sampling frequency of 51.2 kHz. All microphones were calibrated using a pistonphone prior to the measurements.

### 2.4 Measurement parameters

An overview of the measurement parameters is provided in Table 1. Acoustic measurements were taken at 10 different flow speeds (the freestream velocity) between $U_{\infty }=$ 5.4 and 50.8 m/s, corresponding to Reynolds numbers based on chord of $Re_{C}=2.5\times 10^{4}$ to $2.25\times 10^{5}$. Measurements for all symmetric airfoils with both natural and forced transition were taken at an airfoil geometric angle of attack of α=0° to 20°. Cambered airfoils were measured at α=−10° to 20°, where the models were rotated around their half chord location. As shown by Awasthi et al. [4], the airfoil spanwise effective angle of attack distribution can be calculated using Prandtl's lifting-line theory [5] with the geometric angle of attack as input.

### 2.5 Data processing

**Fig. 3:**
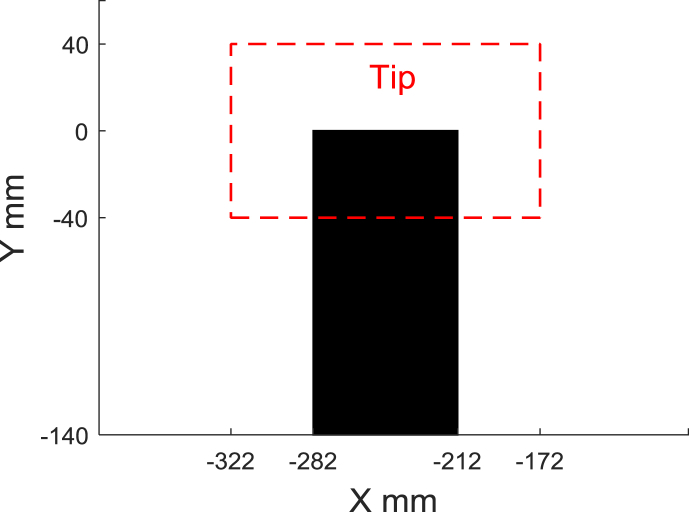
One-third-octave band sound map integration region for the tip (shown in red) of the wall-mounted finite airfoil (shown in black).

**Fig. 4:**
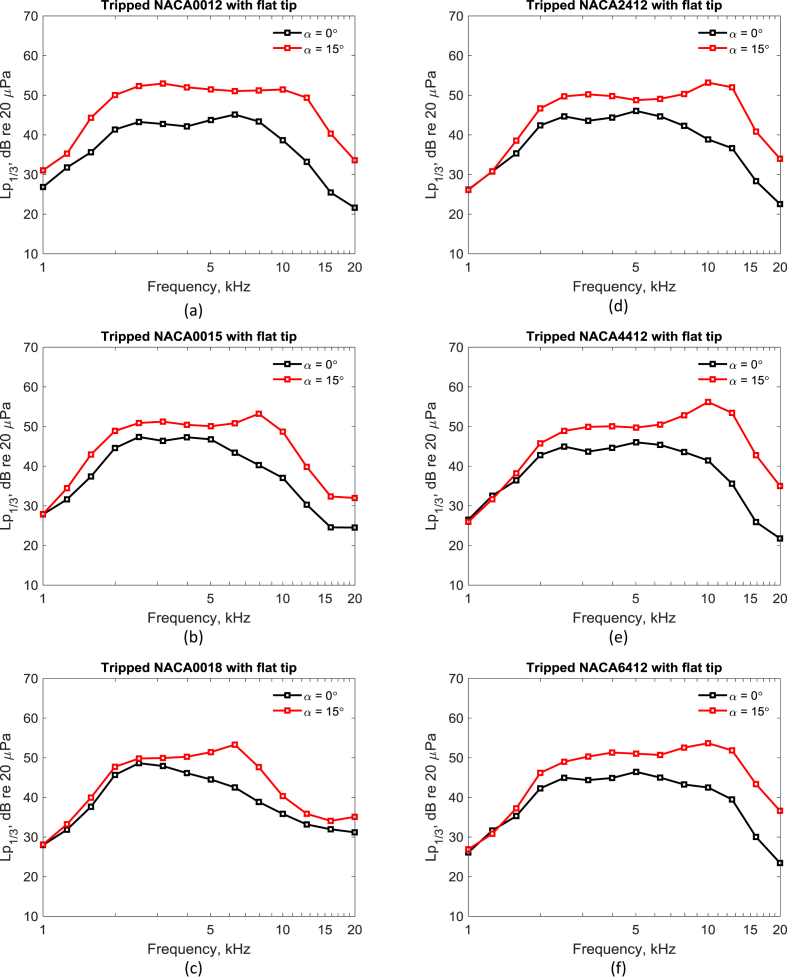
Tip noise spectra for the tripped airfoil with flat tip at α=0° and 15° and $Re_{C}=\;2.25\times 10^{5}$, based on chord. (a) to (c) tripped symmetric NACA0012, NACA0015 and NACA0018 airfoils with flat tip, respectively. (d) to (f) tripped cambered NACA2412, NACA4412 and NACA6412 airfoils with flat tip, respectively.

The microphone time pressure histories were processed using acoustic beamforming [6]. This is a popular method that produces maps of sound source distributions such that the noise sources can be visually observed [7,8]. Acoular was used to process the data. This is a Python module for acoustic beamforming that processes multichannel microphone data acquired in the time domain [6]. The acoustic data acquired with each microphone were transferred to the frequency domain using a Fast Fourier Transformation with blocks of 8192 samples and 50% overlapping using a Hann window. The Cross-Spectral Matrix was then created and set as an input to the beamforming algorithm. Formulation IV [9] was chosen as the steering vector formulation for the beamforming. Ultimately, the CLEAN-SC deconvolution algorithm with diagonal removal was used to remove the influence of the microphone array's point spread function [10,11]. One-third-octave band acoustic spectra were acquired by defining a two-dimensional integration region, where the width of the region is 57% of the span and the length is 214% of the chord (extending from   x=−320 mm, y=−40 mm to x=−170 mm, y= 40 mm) to encompass the airfoil tip region, as shown in Fig. 3. Table 3 provides an example of the integrated one-third-octave band tip noise spectra for a tripped NACA0012 airfoil with flat tip at geometric angles of attack of α=0 :2.5 :15° and 20°, and a Reynolds number based on chord of $Re_{C}=2.25\times 10^{5}$. Examples of tip noise spectra for tripped airfoils with flat and rounded tip at $Re_{C}=2.25\times 10^{5}$, based on chord, are shown in Fig. 4.
